# Bilateral primary ovarian Ewing sarcoma recurring as left submandibular lymphadenopathy diagnosed on cytology

**DOI:** 10.4322/acr.2024.499

**Published:** 2024-07-12

**Authors:** Shaivy Malik, Neha Kawatra Madan, Meetu Agrawal, Rajni Yadav, Adarsh Barwad

**Affiliations:** 1 Vardhman Mahavir Medical College and Safdarjung Hospital, Department of Pathology, New Delhi, India; 2 All India Institute of Medical Sciences, Department of Pathology, New Delhi, India

**Keywords:** Ovary, Cytology, Neoplasm Metastasis, Lymph Nodes, Molecular Conformation

## Abstract

Ewing sarcoma (ES) is a highly malignant and aggressive small round-cell tumor originating from primitive neuroepithelium and mesenchymal stem cells. It is usually seen in children and adolescents with a male predilection and a preponderance to occur in long bones. Although skeletal/soft tissue ES is encountered in clinical practice, primary ES of the genital tract, particularly bilateral primary ovarian ES, is highly uncommon, with only a handful of cases reported worldwide. Ovarian ES is occasionally reported to involve para-aortic and pelvic lymph nodes in advanced stages. Still, cervical lymph node metastasis from ovarian ES is an infrequent clinical occurrence and, when present, indicates a worse prognosis. Here, we present an intriguing case of bilateral peripheral primary ovarian ES in an adult female, recurring as metastasis in the left submandibular lymph node. This case underlines the importance of keeping metastasis from ES as a possible differential while diagnosing metastatic small round cell tumors in peripheral lymph nodes. It also highlights the usefulness of a minimally invasive diagnostic modality of fine needle aspiration cytology and cell block preparation with applied ancillary techniques of immunohistochemistry and confirmatory molecular testing by fluorescence in-situ hybridization (FISH), for an accurate and quick diagnosis of such entities. The cytological diagnosis of our patient helped in the prompt and early initiation of chemotherapy without requiring any invasive procedure.

## INTRODUCTION

Ewing’s Sarcoma (ES) is a highly malignant and aggressive small round cell tumor originating from primitive neuroepithelium and mesenchymal stem cells.^1^ James Ewing^2^ first described ES in the early 1920s, and it still serves as a prototype of the small round blue cell sarcomas of bone. ES is genetically defined according to the most recent 5th edition of the World Health Organization (WHO) Classification of Tumors of Soft Tissue and Bone Tumors. It is an embryonal neoplasm composed of tumor cells showing varying degrees of neuroectodermal differentiation.^3^ ES usually occurs in children and adolescents, accounting for 4-17% of all pediatric soft tissue tumors, and is the second most common primary malignancy of bone in them after osteosarcoma.^4,5^ It is only seldom seen in adults. ES mainly affects Caucasians and Hispanics with a slight male predilection.^6^ It is preponderant to occur in the diaphysis and metaphysis of long bones, pelvis, and ribs. Although skeletal and soft tissue ES are encountered in clinical practice, primary ES of the genital tract, particularly bilateral primary ovarian ES, is highly uncommon with only a handful of cases reported worldwide.^7,8^ Ovarian malignancies are known to involve para-aortic and pelvic lymph nodes in the advanced stages.^9^ Metastatic cervical lymph nodes from ovarian malignancies are rarely seen and, when present, indicate a dismal prognosis. To our knowledge, cervical lymph node metastasis from ovarian ES has never been described before. An extensive and comprehensive literature search was performed on PubMed, Scopus, Web of Science, EMBASE, and Google Scholar using the keywords ovary, ES, lymph node, and metastasis. However, it elicited no reports of ovarian ES metastasis to cervical lymph nodes. Fine needle aspiration cytology (FNAC) has proven useful in diagnosing primary and metastatic small round cell tumors. However, there is scarce literature describing cytomorphological features of metastatic ES for an accurate diagnosis.

Here, we present a very intriguing and extremely rare case of bilateral primary ovarian ES in an adult female. This is an uncommon site, age group, and gender for ES, recurring as distant metastatic deposits in the left submandibular lymph node.

## CASE REPORT

A 33-year-old woman presented with left submandibular swelling of 1.5 months duration. She had no associated upper respiratory or other infectious symptoms. Family history was unremarkable, and there was no history of chronic illness. However, she was submitted to a surgical procedure performed for bilateral ovarian tumors two years back when she was 31 years old. Her records revealed that she underwent a total abdominal hysterectomy with bilateral salpingo-oophorectomy and pelvic and para-aortic lymph node dissection along with omentectomy. Histopathological diagnosis of the resection specimen was confirmed as ES in bilateral ovaries. Both the fallopian tubes, uterus, cervix, para-aortic/ pelvic lymph nodes, and omentum were free of tumor. Post-operatively, she was given 6 cycles of VAC + IE (Vincristine, Adriamycin, Cyclophosphamide, Ifosfamide, and Etoposide) based combination chemotherapy. A subsequent PET-CT scan showed no abnormal ^18^F-fluorodeoxyglucose (FDG) avid lesion indicating no evidence of residual tumor anywhere in the body. She had been disease-free for the past two years before presenting with left submandibular swelling.

**Figure 1:**
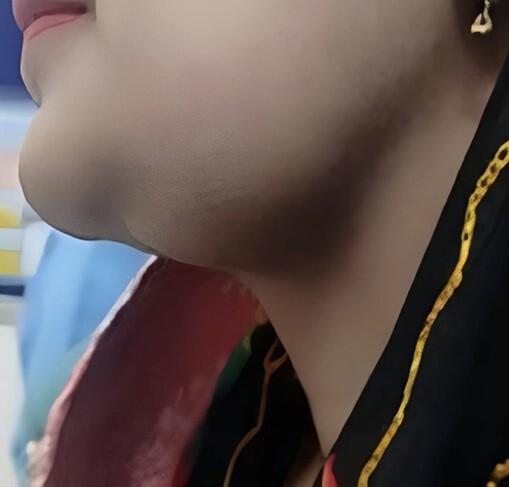
Left submandibular swelling, 4 × 3 cm in size, ill-defined, tender, firm in consistency with restricted mobility.

On examination, the submandibular swelling was a 4 × 3 cm size single lesion, tender, firm in consistency with restricted mobility, and the overlying skin was unremarkable as shown in Figure 1. No other swelling on the same or contralateral side could be palpated. Systemic examination was unremarkable.

FNAC was performed from the swelling under all aseptic precautions. Air-dried May Grunwald-Giemsa (MGG) stained and ethyl alcohol fixed Papanicolaou stained smears were prepared. The cell block was also prepared from the aspirated material using 10% neutral buffered formalin as a fixative. Routine hematoxylin and eosin-stained sections were prepared from the paraffin-embedded material.

**Figure 2:**
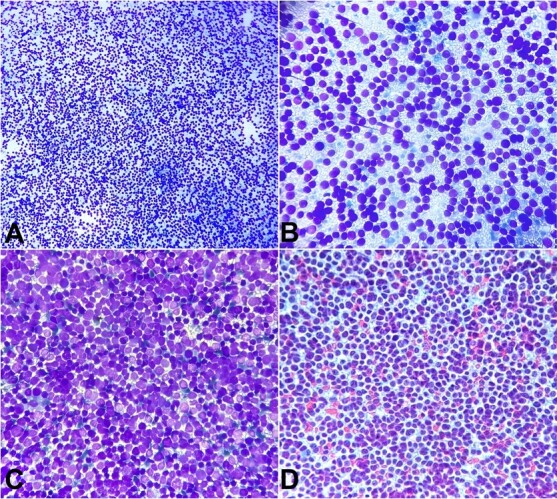
Photomicrographs of the FNAC. **A –** Hypercellular cytological smears showing malignant cells dispersed singly as well as in small loosely cohesive clusters with the formation of occasional rosette-like structures (May Grunwald-Giemsa (MGG) stain ×100); **B –** Malignant cells present in a characteristic tigroid background, (MGG stain ×400); **C –** Two cell populations can be appreciated with small round blue cells having high nuclear to cytoplasmic ratio interspersed with large pale cells with abundant finely vacuolated cytoplasm, (MGG stain ×400); **D –** Small round blue cells with dense nuclei with slightly irregular nuclear membranes, coarse chromatin, and inconspicuous nucleoli. Larger cells have fine granular chromatin with 1-2 small nucleoli, (Papanicolaou stain ×400).

On cytological examination, smears were highly cellular and showed the presence of malignant cells dispersed singly as well as arranged in small, loosely cohesive clusters against a characteristic tigroid background. Occasional rosette-like structures without a fibrillar center were present, shown in Figures 2A and 2B. Two characteristic cytomorphological cell populations were identified. The first one is comprised of small, round blue cells with a high nuclear-to-cytoplasmic ratio, scant cytoplasm, and irregular nuclei with dense, coarse chromatin and inconspicuous nucleoli. Nuclear molding could be appreciated in a few cell clusters as well. These small dark cells were interspersed with a second population of tumor cells comprising large pale cells with abundant cytoplasm and occasional fine vacuoles or clear cytoplasmic spaces corresponding to the large deposits of glycogen. They had round to oval nuclei with fine granular chromatin and 1-2 small nucleoli, as shown in Figures 2C and 2D. Frequent atypical mitoses were identified as well.

**Figure 3:**
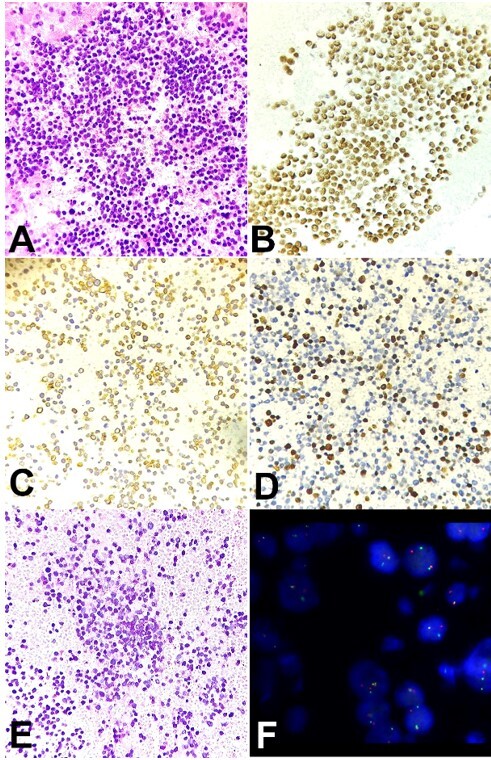
Photomicrographs of the cellblock slices. **A –** Cell block preparation showing malignant cells with similar morphology as seen on smear (H&E ×400); **B –** Tumor cells showing strong nuclear positivity for NKX2.2 (×400); **C –** Tumor cells showing strong and diffuse membranous positivity for CD 99, (×400); **D –** Tumor cells showing around 40% Ki-67 proliferation index, (×400); **E –** Periodic acid-Schiff (PAS) special stain highlighting glycogen present in the larger pale cells with abundant cytoplasm and fine vacuoles, (PAS ×400); **F –** Fluorescence in-situ hybridization (FISH) performed using break-apart probe for EWSR1 gene, showing split green and red signals in the cells, indicating EWSR1 gene rearrangement.

Routine five-micron thick, hematoxylin and eosin-stained sections prepared from formalin-fixed and paraffin-embedded cell block material were cellular and showed the presence of malignant cells scattered singly and in loose cohesive groups with similar morphological features as described on examination of smears as shown in Figure 3A.

Immunohistochemistry (IHC) was performed on two-micron thick sections taken on Poly-L-Lysine coated slides. An extensive panel of immunohistochemical antibodies were applied to clinch an accurate diagnosis. On IHC, the tumor cells showed strong nuclear positivity for NKX2.2, as shown in Figure 3B, a highly sensitive and specific marker for ES, and strong diffuse membranous positivity for CD 99, as shown in Figure 3C. The malignant cells were also diffusely positive for Vimentin, Neuron-specific enolase (NSE), and focally positive for Synaptophysin. The tumor cells were negative for Leucocyte Common Antigen (LCA)/CD 45, pan-Cytokeratin (AE1/AE3), Desmin, MyoD1, Chromogranin, WT-1, SALL4, and OCT3/4. Ki-67 proliferation index was around 40%, as shown in Figure 3D. Periodic acid-Schiff (PAS) special staining was performed, highlighting the cytoplasmic glycogen in the larger cells with abundant pale cytoplasm with fine vacuoles, as shown in Figure 3E. Additionally, a confirmatory molecular cytogenetic study of fluorescence in-situ hybridization (FISH) was performed on the section from cell block preparation using a break-apart probe for the *EWSR1* gene, which revealed split green and red signals in the cells, indicating *EWSR1* gene rearrangement as shown in Figure 3F.

Based on the cytomorphological features and confirmation by ancillary techniques of IHC and molecular cytogenetic modality of FISH, in adjunct with clinical history, a diagnosis of metastatic deposits of ES in the left submandibular lymph node was given. She subsequently underwent an ^18^FDG PET-CT scan to look for any other occult metastasis. However, no other metabolically avid lesion was detected apart from the left submandibular lymph node. The patient was immediately started on VAC + IE-based combination chemotherapy, to which she instantaneously started showing an excellent response, and the cervical lymph node swelling has substantially reduced in size. She has been regularly receiving chemotherapy and has been kept on close follow-up in the medical oncology department.

## DISCUSSION

ES represents a biologically aggressive and poorly differentiated malignant neoplasm composed of small round blue cells and is a key prototype clinical entity and member of the small round blue cell sarcomas of bone, showing varying degrees of neuroectodermal differentiation.^10^ The most recent 5th edition of the World Health Organization (WHO) Classification of Tumors of Soft Tissue and Bone Tumors genetically categorizes small round cell sarcoma of bones into four categories, which are molecularly defined and have distinct clinical outcomes and prognosis despite their overlap in morphological and immunophenotypic features. They include Ewing Sarcoma with *ETS* gene rearrangements, tumors with gene fusions involving *EWSR1-non ETS*, *CIC* rearranged tumors, and sarcomas with *BCOR* genetic alterations.^11^ Conventional ES or Ewing Sarcoma with *ETS* gene rearrangements, Askin tumor (which is a malignant small round cell neoplasm arising in the soft tissue of the thoracic-pulmonary wall), Neuroepithelioma and Peripheral Primitive Neuroectodermal Tumor (which is characterized by the presence of Homer Wright pseudo-rosettes), all belong to the same family of neuroectodermal tumors.^12^ However, the 5^th^ edition of the World Health Organization (WHO) Classification of Tumors of Soft Tissue and Bone Tumors recommends the usage of the terminology Ewing sarcoma as an umbrella term to describe all these neoplasms since they all have similar genetic profiles, clinical characteristics, and prognosis. These tumors harbor identical cytogenetic aberration, which is translocation (11;22) (q24;q12), leading to characteristic *EWSR1::FLI1* (Ewing sarcoma breakpoint region 1/Friend leukemia integration 1) transcript, which is seen most commonly in around 85-90% of the cases.^13^ However, in a small subset of 5% of cases, translocation (21;22)(q22;q12) resulting in *EWSR1::ERG* (ETS-related gene) chimeric transcript is also seen. Rarely, a fusion of the *EWS* gene with other members of the *ETS* (E-26 transformation-specific) family of transcription factors, including *ETV1* (7p22), *ETV4* (17q21), and *FEV* (2q35-36) are seen as well.^14,15^ Round cell sarcomas with *EWSR1-non ETS* gene fusions are defined by *EWSR1::NFATc2, FUS::NFATc2 and EWSR1::PATZ1* molecular alterations. Round cell sarcomas with BCOR alterations usually harbor *BCOR::CCNB3* and *BCOR::MAML3* genetic fusions, and *CIC*-rearranged sarcomas are characterized by *CIC::DUX4* chimeric transcript.

Although ES classically occurs in bone and soft tissues, its occurrence as a primary malignancy at extra-skeletal sites has been only infrequently reported in kidney,^16^ lung,^5^ esophagus,^10^ ileum,^17^ abdominal wall,^18^ pelvis,^19^ thyroid,^20^ penis,^21^ and female genital tract including ovary,^22^ uterus^23^ and cervix.^24^ Fewer than 100 primary neuroectodermal tumors of the ovary have been reported in the literature, and bilateral primary ovarian ES is an even rarer occurrence, with only a handful of cases reported worldwide.

ES usually occurs in children and adolescents, with approximately 50% of cases occurring between 10 and 20 years of age.^25^ It has a definite male preference, with a male-to-female ratio of approximately 1.5:1.^6^ Therefore, bilateral primary ovarian ES occurring in our patient, who is an adult female, is an occasional clinical occurrence.

Ovarian malignancies usually spread by direct extension, transperitoneal, or lymphatic route, and uncommonly by hematogenous route. By the lymphatic route, metastasis is commonly seen in retroperitoneal (pelvic and/or para-aortic) lymph nodes in advanced stages of ovarian cancer, i.e., Stage III, according to the FIGO (International Federation of Gynecology and Obstetrics) Stage. However, lymphatic spread to cervical lymph nodes is seldom seen and has been reported in a mere 4% to 6% of patients with ovarian cancer.^26^ When present, it is classified as distant metastasis, i.e., Stage IV per FIGO staging, and confers a dismal prognostic outcome to the patients. In the series by Dvoretsky et al.^27^ comprising 100 autopsies of female patients who died as a result of ovarian cancer, the incidence of lymphadenopathy in the supraclavicular lymph nodes was found to be only 4%, while none of the cases showed involvement of submandibular lymph nodes.

On performing an extensive literature search as described previously, to our knowledge, bilateral primary ovarian ES recurring as metastatic deposits in unilateral submandibular lymph nodes post-successful treatment is an exceptionally rare clinical occurrence and has never been reported before. Table 1 summarizes the clinical characteristics of previously reported cases of ovarian ES with lymph node involvement.

**Table 1:** Clinical characteristics of previously reported cases of primary Ovarian Ewings Sarcoma with lymph node involvement

**Ref.**	**Primary Site**	**Age (y)**	**Clinical Presentation**	**Involved lymph nodes**	**Treatment and outcome**
Kim et al.^28^	Right ovary	18	Rapidly growing lower abdominal mass. CT revealed 16 × 13 cm-sized solid and cystic multi-lobulated mass in the pelvic cavity.	Pelvic and para-aortic lymph nodes	Right salpingo-oophorectomy, left ovary wedge resection, omentectomy, para-aortic and pelvic lymph node resection and chemotherapy, including taxol/carboplatin, pelvic cavity radiotherapy, followed by vincristine, actinomycin, cyclophosphamide, doxorubicin (VACA). Death after 10 months due to septic shock.
Ateser et al.^29^	Right ovary	28	24th week of pregnancy, a 3-day history of generalized abdominal pain, nausea, and vomiting. Abdominal ultrasound showed a 24-week fetus and a multi-lobulated solid mass situated in the right lower abdominal quadrant extending from the pelvis up to the umbilicus.	Para-aortic Lymph nodes	Right salpingo-oophorectomy followed by chemotherapy including doxorubicin, cyclophosphamide, vincristine. The patient delivered at 37 weeks of gestation. She had extensive metastatic disease and was treated with chemotherapy and radiotherapy post-delivery. However, she declined further therapy and died due to progressive disease 13 months after the initial diagnosis.
Kuk et al.^30^	Right ovary	32	Ovarian PNET was detected during her second Cesarean section. She had a past history of oophorectomy due to a huge mature solid teratoma in left ovary during her first Cesarean section.	Bilateral internal iliac lymph nodes	Right salpingoophorectomy and peritoneal mass excisional biopsy were performed followed by 8 cycles of multiple regimen chemotherapy with vincristine, doxorubicin, cyclophosphamide, and etoposide, ifosfamide (VDC/IE). She remained in complete remission with no evidence of disease 54 months from presentation.
Ahadi et al.^31^	Left ovary	30	Pelvic pain and distention for less	Para-aortic Lymph nodes	Laparotomy for resection of tumor followed by Bleomycin, Cisplatin, and Etoposide based chemotherapy. However, she died two months later due to extensive metastatic disease before completing all the cycles of chemotherapy.
			than two weeks. Abdominopelvic CT scan revealed an irregular heterogeneous mass in the left adnexa with adhesion to the surrounding organs.		
Chao et al.^22^	Right ovary	67	Abdominal distention and changes in bowel habits over the previous 4 months. Ultrasonography revealed a pelvic mass of approximately 22 cm in diameter. Colonoscopy indicated sigmoid colon adhesions and stenosis	Lymph nodes around the colon	Tumor cytoreductive surgery with right salpingo-oophorectomy, appendectomy, infracolic omental excision, and partial excision of the sigmoid colon and small intestine. The patient refused further treatment and died 6 months after her initial symptoms.
Index Case	Both ovaries	33	Left submandibular swelling of 1.5 months duration. History of bilateral ovarian PNET treated successfully with bilateral salpingo-oophorectomy and total hysterectomy followed by chemotherapy and was disease free for 2 years.	Metastatic recurrence as left submandibular lymphadenopathy	Patient immediately started on VAC + IE based combination chemotherapy following cytological diagnosis and has shown excellent response. She is currently receiving chemotherapy and has been kept on close follow-up.

Ref. = reference; y = years.

This case also highlights the vital role of a simple, inexpensive, non-invasive, and yet easy-to-perform diagnostic modality of fine-needle aspiration cytology in diagnosing such entities. Cytomorphological features, along with cell block preparation and ancillary techniques of IHC and molecular diagnosis by FISH, aided in prompt diagnosis of metastatic recurrence of ES and early initiation of chemotherapy in the patient without requiring an invasive procedure like a core needle or an excisional biopsy. Owing to the presence of malignant small round cells against a characteristic tigroid background along with atypical mitosis helped in arriving at a possible differential diagnosis, which included ES, germ cell tumor, i.e., dysgerminoma, and rhabdomyosarcoma. These neoplasms produce a peculiar background on air-dried smears described as a tigroid background. It is characterized by a relatively granular, reticulated material that Lopes-Cardozo described as “foamy, lazy, tiger-striped or astrakhan”^32^. It occurs as a result of the disruption of the fragile glycogen-containing cytoplasm of neoplastic cells during smearing.

Cellblock, and IHC further play a key role in arriving at an accurate diagnosis. An extensive panel of immunohistochemical markers applied which included LCA, pan-CK, Vimentin, CD 99 (also called MIC2/T cell surface glycoprotein E2 or p30/32 protein),^33^ NKX2.2, SALL4, OCT3/4, Desmin, MyoD1, Synaptophysin, Chromogranin, NSE, and Ki-67 helped in ruling out the differentials and arriving at a definite diagnosis. Negative SALL4 and OCT3/4 ruled out the possibility of a germ cell tumor, like dysgerminoma. The malignant cells being negative for Desmin and MyoD1 negated the possibility of rhabdomyosarcoma, and negativity for LCA helped rule out lymphoma. Also, the tumor cells were pan-CK negative, which helped rule out metastatic carcinoma. Moreover, unequivocal strong diffuse membranous CD 99 positivity along with strong nuclear NKX2.2 positivity as well as diffuse cytoplasmic positivity for Vimentin and NSE and focal positivity for Synaptophysin helped in clinching the diagnosis of metastatic deposits of ES in the submandibular lymph node. NKX2.2 is a relatively new and valuable marker for ES, with a sensitivity of 93% and a specificity of 89%, and aids in the differential diagnosis of small round blue cell neoplasms.^[30]^ The NKX2.2 gene was recently identified as a target of *EWSR1::FLI-1*, the fusion protein specific to ES, and was shown to be differentially upregulated in ES based on array-based gene expression analysis. Additionally, a positive combination of NKX2.2 and CD 99 further enhances the specificity in diagnosing ES. Traditionally, CD 99 and FLI-1 antibodies have been used for diagnosing ES. However, their accuracy has been controversial.^34^ Therefore, NKX2.2, along with CD 99, is a useful combination to diagnose ES. Further, a confirmatory diagnosis can be made with the help of molecular studies and cytogenetics using fluorescence in-situ hybridization (FISH) demonstrating *EWSR1* gene rearrangement, as was done in our case.

ES is a group of highly malignant and notorious tumors with rapid tumor progression and poor prognosis. The treatment for ES includes surgical resection, chemotherapy, and radiotherapy. However, despite this multi-modal therapeutic approach, overall survival for the patients remains low, as reported in studies by Mulsow et al.^35^ and Abboud et al.^36^ Kuk et al.^30^ reported an overall survival in ovarian ES patients ranging from 10.8 to 36 months. Moreover, owing to the rarity of ovarian ES, standard effective treatment guidelines do not exist. The most successfully employed approach includes surgical reduction of the tumor with a staging laparotomy (including unilateral or bilateral salpingo-oophorectomy with or without total hysterectomy and pelvic lymph node dissection and omentectomy and any other adjacent tissue excision depending on the extent of involvement and surgical feasibility) followed by postoperative adjuvant chemotherapy. VACD (Vincristine, Actinomycin, Cyclophosphamide, Doxorubicin) combined with IE (Ifosfamide, Etoposide) has substantially improved the 5-year tumor-free survival and overall rate with fewer adverse effects.^37^ Due to the aggressive nature of this neoplasm, the risk of residual disease and relapse persists despite the effective administration of therapy, as was seen in our patient as well. Moreover, distant metastasis reflects an even worse clinical prognosis.

## CONCLUSION

This case underlines the importance of keeping metastasis from ES as a differential while diagnosing metastatic small round cell tumors in the lymph node despite their exceptionally rare occurrence. It also highlights the usefulness of a simple and minimally invasive procedure of FNAC, along with cell block preparation and ancillary techniques of immunohistochemistry and molecular cytogenetics, in an accurate and timely diagnosis of such entities. The cytological diagnosis helped in prompt diagnosis and early initiation of chemotherapy in metastatic ES recurrence without requiring any invasive procedure.
